# COVID-19 vaccination recommendations and practices for women of reproductive age, U.S. Physicians, Fall 2021

**DOI:** 10.1016/j.pmedr.2023.102141

**Published:** 2023-02-13

**Authors:** Mehreen Meghani, Lauren B. Zapata, Kara Polen, Romeo R. Galang, Hilda Razzaghi, Dana Meaney-Delman, Sascha Ellington

**Affiliations:** a Centers for Disease Control and Prevention Foundation, Atlanta, GA, United States; b Division of Reproductive Health, Centers for Disease Control and Prevention, Atlanta, GA, United States; c Division of Birth Defects and Infant Disorders, Centers for Disease Control and Prevention, Atlanta, GA, United States; d Immunization Services Division, Centers for Disease Control and Prevention, Atlanta, GA, United States

**Keywords:** Primary care physicians, COVID-19 vaccination, Pregnant women, Reproductive health, DocStyles

## Abstract

Pregnant people with COVID-19 are at increased risk for severe illness and adverse pregnancy outcomes. COVID-19 vaccinations are safe and effective, including for pregnant and recently pregnant people. The objective of this analysis was to describe the extent to which primary care physicians across the United States report confidence in talking with female patients of reproductive age about COVID-19 vaccination, recommending COVID-19 vaccinations to pregnant patients, and offering COVID-19 vaccinations at their practices in fall 2021.

We analyzed cross-sectional data from the Fall 2021 DocStyles survey, a web-based panel survey of U.S. primary healthcare providers (64% response rate). Family practitioners/internists, obstetrician-gynecologists, and pediatricians were asked about confidence in talking with female patients of reproductive age about COVID-19 vaccination, vaccination practices regarding pregnant patients, and offering COVID-19 vaccinations. We describe results overall and by select physician characteristics. Among 1501 respondents, most were family practitioners/internists (67%), 17% were obstetrician-gynecologists, and 17% were pediatricians. Overall, 63% were very confident talking with female patients of reproductive age about COVID-19 vaccination, 80% recommended pregnant patients get vaccinated as soon as possible, and 50% offered COVID-19 vaccinations at their current practice. Obstetrician-gynecologists were most confident in talking with female patients, but only one-third offered the vaccine at their practices. This analysis found that most physicians felt confident talking about COVID-19 vaccinations and recommended pregnant patients get vaccinated as soon as possible. Provider recommendation for vaccination remains a key strategy for achieving high vaccination coverage, and consistent recommendations may improve vaccine acceptance among pregnant and postpartum people.

## 1 Introduction

Pregnant and recently pregnant people are at increased risk for severe illness from COVID-19. Pregnant people are also at increased risk for adverse pregnancy outcomes such as preterm birth and stillbirth (Woodworth et al., 2020, Allotey et al., 2019, Centers for Disease Control and Prevention, 2022, DeSisto et al., 2021). COVID-19 vaccines were available to pregnant people who were eligible as of December 2020, and vaccination was strongly recommended for all pregnant people as of August 11, 2021 (Centers for Disease Control and Prevention, Centers for Disease Control and Prevention, 2022). During August–November 2021, vaccine coverage among pregnant women was 45.1 %, substantially lower than among non-pregnant women of reproductive age (64.9 %) (Razzaghi et al., 2022). Vaccination during pregnancy is safe and effective and reduces the risk of severe illness and adverse outcomes from COVID-19 (Prasad et al., 2022). Healthcare providers have a unique opportunity to counsel their pregnant and recently pregnant patients on COVID-19 vaccination, improve confidence, and encourage uptake (Centers for Disease Control and Prevention, 2021). Prior studies have shown that healthcare providers are among the most trusted sources for information on vaccines. A study on provider recommendation for COVID-19 found that provider recommendation was associated with higher likelihood of getting vaccinated, as well as higher likelihood of having confidence that vaccines are safe and effective (Nguyen et al., 2021). We present findings from a web-based panel survey of health care providers who were asked about confidence in talking with female patients of reproductive age about COVID-19 vaccination, vaccination practices regarding pregnant patients, and offering COVID-19 vaccinations.

## 2 Methods

We analyzed cross-sectional data from the fall 2021 DocStyles survey (administered September 14–November 10, 2021), a web-based non-probability panel survey of U.S. healthcare providers commissioned by Porter Novelli Public Services (http://styles.porternovelli.com). DocStyles participants are recruited from SERMO’s (http://www.sermo.com) global medical panel, which comprises 350,000 panelists who are verified using a double opt-in sign up process with telephone confirmation at place of work. Respondents were sampled and quotas were determined to reach 1,000 family practitioners or internists, 250 obstetrician-gynecologists, 250 pediatricians, and 250 nurse practitioners or physician assistants. At the time of the survey, respondents only practiced in the United States, were actively seeing patients, and had been practicing for at least 3 years. Respondents surveyed were also providing care to women of reproductive age (female patients 15–49 years). Participation was voluntary, and respondents received an honorarium that ranged from $26 to $68 depending on how many questions were asked. The survey was designed to describe healthcare provider attitudes and practices on a variety of health topics, including COVID-19 vaccination recommendations and practices. The COVID-19 vaccination-related questions were fielded with primary healthcare physicians including family practitioners/internists, obstetrician-gynecologists, and pediatricians. Physicians were asked about their confidence level talking with female patients of reproductive age about COVID-19 vaccination, including pregnant and postpartum patients, whether they recommended COVID-19 vaccination during pregnancy, and if their practice currently offered COVID-19 vaccinations. We described characteristics of respondents by type of primary healthcare physician and described COVID-19 vaccination practices by select physician characteristics and provider type. We used Chi-square tests of independence to determine differences among groups relating to COVID-19 vaccination practices, with *p* < 0.05 considered statistically significant. We analyzed the data using SAS software, version 9.4 (SAS Institute Inc, Cary, NC). This activity was reviewed by Centers for Disease Control and Prevention (CDC) and was conducted consistent with applicable federal law and CDC policy.^1^

## 3 Results

A total of 1,501 physicians completed the survey (64 % response rate). Most were family practitioners/internists (67 %), 17 % were obstetrician-gynecologists, and 17 % were pediatricians. Most worked primarily in group outpatient settings (67 %), had practiced medicine for >10 years (67 %), were male (64 %), saw between 51–100 patients per week (55 %), and saw ≥1 pregnant patients per week (64 %). All US census regions were represented (33 % South, 23 % Northeast, 21 % Midwest, and 22 % West). Respondent characteristics varied by physician type. Approximately 52 % of obstetrician-gynecologists and pediatricians were female compared with 28 % of family practitioners/internists. Additionally, 53 % of obstetrician-gynecologists had been practicing for more than 20 years compared with 40 % of family practitioners/internists and pediatricians (Table 1).

**Table 1:** Characteristics for Total Respondent Group and Type of Physician – Fall DocStyles, United States, 2021 (*N* = 1,501).

Physician Characteristic	Total (*N =* 1,501)	Family Practitioner/Internist (*n* = 1,000)	Pediatrician (*n* = 251)	Obstetrician-Gynecologist (*n* = 250)
	*N* (%)	*N* (%)	*N* (%)	*N* (%)
**Age (median)**	49	48	48	51
**Gender**^a^				
Female	542 (36 %)	282 (28 %)	129 (51 %)	131 (52 %)
Male	957 (64 %)	716 (72 %)	122 (49 %)	119 (48 %)
**Number of patients per week**				
1 to 50	243 (16 %)	153 (15 %)	46 (18 %)	44 (18 %)
51 to 100	827 (55 %)	548 (55 %)	137 (55 %)	142 (57 %)
101 to 200	363 (24 %)	248 (25 %)	59 (24 %)	56 (22 %)
201 to 500	68 (4 %)	51 (5 %)	9 (4 %)	9 (3 %)
**Numbers of pregnant patients per week**				
None	536 (36 %)	341 (34 %)	169 (67 %)	26 (10 %)
≥1	965 (64 %)	659 (66 %)	82 (33 %)	224 (90 %)
**Number of years practicing**				
<10	350 (23 %)	253 (25 %)	47 (19 %)	50 (20 %)
10 to 19	482 (32 %)	319 (32 %)	96 (38 %)	67 (27 %)
≥20	669 (35 %)	428 (43 %)	108 (43 %)	133 (53 %)
**Primary work setting**				
Individual outpatient practice	241 (16 %)	169 (17 %)	23 (9 %)	49 (20 %)
Group outpatient practice or clinic	1002 (67 %)	652 (65 %)	180 (72 %)	170 (68 %)
Inpatient practice/hospital	258 (17 %)	179 (18 %)	48 (19 %)	31 (12 %)
**Census region**				
Northeast	349 (23 %)	232 (23 %)	66 (26 %)	51 (20 %)
Midwest	321 (21 %)	216 (22 %)	53 (21 %)	52 (21 %)
South	501 (33 %)	324 (32 %)	85 (34 %)	92 (37 %)
West	330 (22 %)	228 (23 %)	47 (19 %)	55 (22 %)

a Two primary care physicians did not report gender.

Overall, 63 % of physicians reported being very confident in talking with female patients of reproductive age about COVID-19 vaccination, but 9 % were not confident in talking with female patients, and confidence varied by physician type. Among obstetricians-gynecologists, 88 % were very confident compared with 62 % of pediatricians and 57 % of family practitioners/internists (*p* < 0.05). Confidence also varied by number of pregnant patients physicians saw per week. Among physicians who saw zero pregnant patients per week, 57 % were very confident compared with 66 % of physicians who saw ≥ 1 pregnant patients per week (*p* < 0.05). Most physicians (80 %) recommended pregnant patients get vaccinated as soon as possible; however, this varied by physician type. Among obstetricians-gynecologists, 89 % recommended pregnant patients get vaccinated as soon as possible, compared with 82 % of pediatricians and 77 % of family practitioners/internists (*p* < 0.05) (Table 2).

**Table 2:** Associations between COVID-19 vaccination attitudes and practices and physician characteristics – Fall DocStyles, United States, 2021.

**How confident are you talking to female patients of reproductive age, including pregnant and postpartum patients, about COVID-19 vaccination?**															
		**Physician Type** **(p < 0.0001)**			**Census region**				**Years in practice**			**Pregnant patients per week** **(p < 0.0001)**		**Age**	
	Total (*n* = 1,501)	Family Practitioner/Internist (*n* = 1,000)	Pediatrician (*n* = 251)	Ob/Gyn (*n* = 250)	Northeast (*n* = 349)	Midwest (*n* = 321)	South (*n* = 501)	West (*n* = 330)	<10 (*n* = 350)	10 to 19 (*n* = 482)	≥20 (*n* = 669)	Zero (*n* = 536)	≥1 (*n* = 965)	<50 (*n* = 797)	≥50 (*n* = 704)
Very confident	947 (63 %)	572 (57 %)	155 (62 %)	220 (88 %)	214 (61 %)	205 (64 %)	323 (64 %)	205 (62 %)	205 (59 %)	302 (63 %)	440 (66 %)	307 (57 %)	640 (66 %)	485 (61 %)	462 (66 %)
Somewhat confident	425 (28 %)	322 (32 %)	77 (31 %)	26 (10 %)	101 (29 %)	97 (30 %)	126 (25 %)	101 (31 %)	109 (31 %)	142 (29 %)	174 (26 %)	156 (29 %)	269 (28 %)	236 (30 %)	189 (27 %)
Not very confident	100 (7 %)	82 (8 %)	15 (6 %)	3 (1 %)	28 (8 %)	15 (5 %)	39 (8 %)	18 (5 %)	27 (8 %)	26 (5 %)	47 (7 %)	54 (10 %)	46 (5 %)	55 (7 %)	45 (6 %)
Not at all confident	29 (2 %)	24 (2 %)	4 (2 %)	1 (0 %)	6 (2 %)	4 (1 %)	13 (3 %)	6 (2 %)	9 (3 %)	12 (2 %)	8 (1 %)	19 (4 %)	10 (1 %)	21 (3 %)	8 (1 %)

**Please choose the statement that most closely matches your current practice.**^b^															

		**Physician type** **(p < 0.05)**			**Census region**				**Years in practice**			**Pregnant patients per week**		**Age**	

	Total (*n* = 965)	Family practitioner/Internist (*n* = 659)	Pediatrician (*n* = 82)	Ob/Gyn (*n* = 224)	Northeast (*n* = 228)	Midwest (*n* = 199)	South (*n* = 317)	West (*n* = 221)	<10 (*n* = 226)	10 to 19 (*n* = 330)	≥20 (*n* = 409)	Zero N/A	≥1 N/A	<50 (*n* = 536)	≥50 (*n* = 429)
I recommend pregnant patients get vaccinated as soon as possible	771 (80 %)	505 (77 %)	67 (82 %)	199 (89 %)	179 (79 %)	158 (79 %)	251 (79 %)	183 (83 %)	188 (83 %)	263 (80 %)	320 (78 %)	N/A	N/A	435 (81 %)	336 (78 %)
I recommend pregnant patients wait until the 2nd or 3rd trimester to get vaccinated	129 (13 %)	99 (15 %)	10 (12 %)	20 (9 %)	32 (14 %)	27 (14 %)	44 (14 %)	26 (12 %)	28 (12 %)	45 (14 %)	56 (14 %)	N/A	N/A	71 (13 %)	58 (14 %)
I recommend pregnant patients wait until after delivery to get vaccinated	41 (4 %)	34 (5 %)	3 (4 %)	4 (2 %)	10 (4 %)	7 (3 %)	15 (5 %)	9 (4 %)	6 (3 %)	13 (4 %)	22 (5 %)	N/A	N/A	20 (4 %)	21 (5 %)
I don’t recommend pregnant patients get vaccinated during or after pregnancy	24 (2 %)	21 (3 %)	2 (2 %)	1 (0 %)	7 (3 %)	7 (3 %)	7 (2 %)	3 (1 %)	4 (2 %)	9 (3 %)	11 (3 %)	N/A	N/A	10 (2 %)	14 (3 %)

**Does your practice currently offer COVID-19 vaccinations?**															

		**Provider type** **(p < 0.0001)**			**Census region** **(p < 0.0001)**				**Years in Practice** **(p < 0.0001)**			**Pregnant patients per week**		**Age** **(p < 0.0001)**	

	Total (*n* = 1,501)	Family practitioner/Internist (*n* = 1000)	Pediatrician (*n* = 251)	Ob/Gyn (*n* = 250)	Northeast (*n* = 349)	Midwest (*n* = 321)	South (*n* = 501)	West (*n* = 330)	<10 (*n* = 350)	10 to 19 (*n* = 482)	≥20 (*n* = 669)	Zero (*n* = 536)	≥1 (*n* = 965)	<50 (*n* = 797)	≥50 (*n* = 704)
Yes	743 (50 %)	534 (53 %)	131 (52 %)	78 (31 %)	133 (38 %)	183 (57 %)	242 (48 %)	185 (56 %)	201 (57 %)	255 (53 %)	287 (43 %)	248 (46 %)	495 (51 %)	451 (57 %)	292 (41 %)
No	743 (50 %)	457 (46 %)	115 (46 %)	171 (68 %)	214 (61 %)	132 (41 %)	256 (51 %)	141 (43 %)	145 (41 %)	221 (46 %)	377 (56 %)	285 (53 %)	458 (48 %)	336 (42 %)	407 (58 %)
Not sure	15 (1 %)	9 (1 %)	5 (2 %)	1 (0 %)	2 (1 %)	6 (2 %)	3 (1 %)	4 (1 %)	4 (1 %)	6 (1 %)	5 (1 %)	3 (1 %)	12 (1 %)	10 (1 %)	5 (1 %)

Note: all percentages are column percentages. Ob/Gyn = Obstetrician-Gynecologist. N/A = Not applicable.

^a^Chi-square test for independence.

b This question was asked if the number of pregnant patients seen per week was greater than 0.

Half of the physicians offered COVID-19 vaccinations in their practice at the time of the survey; this varied by physician type, age, census region, and years in practice. Among family practitioners/internists and pediatricians, about half offered COVID-19 vaccinations, while only 31 % of obstetricians-gynecologists offered the vaccine at their practice (*p* < 0.05). More providers aged < 50 years (57 %) offered COVID-19 vaccination compared with providers aged ≥ 50 years (41 %) (*p* < 0.05). By region, 57 % of physicians in the Midwest, 56 % in the West, 48 % in the South, and 38 % in the Northeast offered COVID-19 vaccination in their practice (*p* < 0.05). Among physicians practicing for < 10 years, 57 % offered COVID-19 vaccination in their practice. Among those practicing for 10–19 years, 53 % offered COVID-19 vaccination in their practice, while among those practicing for ≥ 20 years, 43 % offered COVID-19 vaccination in their practice (*p* < 0.05) (Table 2).

## 4 Discussion

In this study, the majority of respondents were very confident discussing vaccination with female patients of reproductive age, with obstetrician-gynecologists being the most confident. This could be attributed to their familiarity with clinical and public health recommendations promoting COVID-19 vaccination to female patients of reproductive age, as well as their experience with other vaccinations commonly given during women’s health visits. This finding is consistent with other surveys on provider attitudes and vaccine practices. In a 2012 DocStyles survey, obstetricians-gynecologists were more likely to recommend the HPV vaccine to patients aged 9–26 years compared with other provider types (Berkowitz et al., 2015). Consistent with CDC recommendations that vaccinations be recommended regardless of gestational age (Centers for Disease Control and Prevention, 2022), in this study most providers reported that they recommend pregnant patients get the COVID-19 vaccination as soon as possible. However, 20 % of the providers recommended that the pregnant patients either delay vaccination or not get vaccinated without the lack of any scientific evidence that delay is warranted, and it remains important that healthcare providers recommend that all pregnant and postpartum patients get vaccinated.

Despite providers’ confidence level, only 50 % offered COVID-19 vaccination at their current practices during fall 2021. The percentage was lowest among obstetricians-gynecologists with less than a third reporting they provided COVID-19 vaccinations at the time of the survey. The reasons for this are likely multifactorial. Vaccines were initially distributed through pharmacies and health departments with secondary distribution to clinics, hospital systems, and physician offices. In addition, some outpatient clinical practices may not be equipped to handle the requirements of cold storage (Goralnick et al., 2021). Greater distribution of vaccines to settings administering outpatient care may increase vaccination rates among individuals who are receptive to receiving the vaccination but who did not have the opportunity to receive vaccination from their primary care provider (Ratzan et al., 2021).

While mounting evidence on COVID-19 vaccines demonstrates the safety and effectiveness of vaccination during pregnancy, rates of COVID-19 vaccination among pregnant people remain lower than for non-pregnant women (Prasad et al., 2022, Razzaghi et al., 2022). Provider recommendations of a vaccination remains a strong predictor of receipt of a vaccination, regardless of type of vaccine, although other factors, such as concerns about vaccine safety among pregnant people and persistent structural inequities that disproportionately affect racial and ethnic minority groups, remain a barrier to vaccination (Brewer et al., 2021, Huddleston et al., 2022, Nguyen et al., 2021, Razzaghi et al., 2020, Yuen and Tarrant, 2020). Moreover, vaccine hesitancy, not limited to COVID-19 vaccination (Razzaghi et al., 2020), is common among pregnant people and common reasons reported by pregnant people for not receiving recommended vaccinations include concerns about safety risks to themselves or their infants, side effects, and effectiveness of vaccines (Huddleston et al., 2022). Healthcare providers of all specialties need to be equipped with up-to-date information about the safety and effectiveness of vaccination during all trimesters of pregnancy to ensure that there are consistent evidence-based recommendations from all segments of the healthcare system.

This study is subject to three limitations. First, DocStyles is a voluntary opt-in panel survey, and sampling is not population-based or random. Therefore, findings may not be generalizable to the US population of primary care physicians, obstetrician-gynecologists, or pediatricians. Second, survey data are self-reported, and responses may be inaccurate due to recall, social desirability, or other reporting errors. Third, data is from fall 2021 and may not reflect current knowledge and practices.

## 5 Conclusion

The overall safety and effectiveness of COVID-19 vaccinations including for pregnant people is well-described (Prasad et al., 2022). This survey found that three in five physicians felt confident discussing the COVID-19 vaccine and four in five physicians recommended that their pregnant patients get vaccinated as soon as possible; however, only 50 % of respondent practices offered COVID-19 vaccine at the time of the survey. Although most providers felt confident, there was variation among the different providers recommending the vaccine during pregnancy. Since provider recommendation for vaccination is strongly associated with patient acceptance and uptake of vaccine, it is important that all healthcare providers consistently discuss and recommend the COVID-19 vaccine to their female patients of reproductive age, including pregnant and postpartum patients.

## Role of the Funder/Sponsor

The Centers for Disease Control and Prevention participated in the design and conduct of the study; collection, management, analysis, and interpretation of the data; preparation, review, or approval of the manuscript; and decision to submit the manuscript for publication.

## Disclaimer

The findings and conclusions in this report are those of the authors and do not necessarily represent the official position of the Centers for Disease Control and Prevention. Mention of a product or company name is for identification purposes only and does not constitute endorsement by the Centers for Disease Control and Prevention.

## Author Contributions

Meghani had full access to all of the data in the study and takes responsibility for the integrity of the data and the accuracy of the data analysis. Concept and design: All authors. Acquisition, analysis, or interpretation of data: All authors. Drafting of the manuscript: Meghani, Zapata, Ellington. Critical revision of the manuscript for important intellectual content: all authors. Statistical analysis: Meghani. Supervision: Ellington.

## Funding/Support

This study was funded by the Centers for Disease Control and Prevention.

## Declaration of Competing Interest

The authors declare that they have no known competing financial interests or personal relationships that could have appeared to influence the work reported in this paper.

## Data Availability

Data will be made available on request.

## Footnotes

1 See e.g., 45C.F.R. part 46; 21C.F.R. part 56; 42 U.S.C. §241(d), 5 U.S.C. §552a, 44 U.S.C. §3501 et seq.
